# Some Considerations Pertaining to Dental Diagnosis

**Published:** 1903-05

**Authors:** John C. Curry

**Affiliations:** Philadelphia


### SOME CONSIDERATIONS PERTAINING TO DENTAL DIAGNOSIS.[1]

BY JOHN C. CURRY, D.D.S., PHILADELPHIA.

In his work on "Oral Surgery" the late Professor Garretson closes his chapter on diagnosis with this remark: "Complexity resolves itself into simplicity through understanding; to see into a pot requires never anything but the lifting of the lid." Understanding is arrived at by deductions based on study and observation. "I do not know . . . I will investigate," says Pasteur.

[1] Read before the Academy of Stomatology, Philadelphia, December 23, 1902.

"The process" (of diagnosis), says Butler, "is thus seen to consist of two elements,—observation in its broadest sense, and reasoning applied to the results of the observation."

To determine the nature of a pathological condition is the first step in the treatment of disease, whether it be apparent or obscure. Without knowledge, all cases are obscure, and even with understanding not all are easy of solution, but most of them are possible.

Some one has said, "Poets are born, not made." So are diagnosticians, with this difference: Poets are capable of expressing what they themselves feel, while the diagnostician must be able to tell what *others* feel, and why, which, after all, is not so difficult a matter if one is able to take into account the various considerations pertaining to a given case.

Diagnosis may be subjective or objective, or both. Subjective considerations are those arrived at by interrogation. Objective considerations are those depending on observation. Among the subjective considerations we note seat of pain, character of pain, length of time it has been present, history of previous trouble, if any, and family history. Objective considerations would include age, sex, state of health, occupation and habits, temperament and idiosyncrasy, and accident. Subjective considerations alone are least to be depended upon in making an accurate diagnosis.

When a patient presents for treatment complaining of pain, the first question naturally is, Where is your pain, and what is its character? and in nine cases out of ten the answer will depend more upon the temperament and condition of the patient than the nature of the pain.

The late Dr. Bonwill seems to have recognized this more fully than any other dentist whom I have known, for he usually insisted on his patient keeping silent while he made the diagnosis. Objectively, I think, however, most diagnosticians will agree that this method is extreme, for by interrogation we can usually get some clue on which to base further investigation.

The point I would make is that in some cases we must make a liberal allowance, while in others we should encourage the patient to describe fully his pain or inconvenience; for some patients, without intending to deceive, magnify their sufferings beyond their true proportion, in order to gain our sympathy; others, desiring to be heroic, try to "grin and bear it" without a murmur; while still another class will suffer to the utmost rather than complain, for fear of having it thought of them that they are making a bid for sympathy. These last-named patients belong to the highly organized, nervous temperaments, sensitive alike in mind and body, slow to bestow their confidence, but unwavering in their trust when once it is given. These are the patients who should be encouraged to speak freely, for they never deceive. They are the leaven of a dental practice.

All pains in or about the teeth are usually spoken of by the patient under the general term of "toothache," and may be described as "jumping," being almost invariably found in connection with exposed pulps, either spontaneous or the result of mechanical or chemical irritation or thermal shock. A dull, heavy pain, with crisis on taking warm foods or liquids, with sense of numbness or fulness, is due to pulp congestion, usually found in vital teeth having large fillings placed too near the pulp. The pain is generally reflected to the teeth of the opposite jaw, or transferred to the ear or supraorbital region, with very slight tenderness on tapping, but not to steady pressure, though considerable force be used. Throbbing, pulsing, and lancinating pains, with tenderness to touch, indicate formation of pus, either in true alveolar abscess, phagedenic pericementitis, or abscess due to presence of foreign bodies, such as broken tooth-picks, forgotten ligatures, etc.

The uneasy, ill-defined pain, not definitely located unless the spot is actually touched, gives us at once a clue to sensitive dentine. I have known a spot of dentine no larger than a pin-head to cause neuralgic twinges that were as hard to account for as they were to endure. These conditions are especially common in strawberry and tomato seasons.

The pain, usually described as neuralgic, sharp, lancinating, intermittent, remittent, or periodic, direct, reflected, or transferred, unless accounted for objectively, is usually associated with the more obscure troubles, such as exostosis, absorption of permanent roots, or pulp-nodules, or they may sometimes be found in senile caries with exposed pulps.

All these varieties of pain vary in character and intensity according to the severity of the case and the temperament of the patient.

Family history may not have so important a place in dental diagnosis as it has in a medical diagnosis, but it may be of value in tracing constitutional tendencies, such as lithiasis, hemorrhagia, recession of gums, abnormalities of dentition, pathological dentition, etc.

Age considerations predispose to or modify many pathological conditions from infancy to senility. Pathological first dentition is a subject on which a volume might be written. Unfortunately for all concerned, the dentist is seldom consulted in these troubles, but, thanks to what must be a special Providence, the rate of mortality is only about three times as high during first dentition as at any other period of life up to sixty years. It may be well for us to remember, however, that besides the ordinary troubles of first dentition, rickets, cretinism, and infantile scorbutus markedly retard or complicate the process of first dentition.

Pathological dentition of the permanent denture is usually confined to the third molar, the eruption of which often gives rise to troubles varying in degree from slight pain and stiffness of the maxillary articulation to crisis and trismus. The subjective symptoms are of such character as to be easily recognized if occurring at the age when the third molars are usually erupted, and are described as "uneasy pains" in the jaw, often reflected to the ear, stiffness of the joint, pain and difficulty in swallowing, and increased salivation.

In a crowded lower jaw with a short angle the third molar may erupt in such a position as to present its occlusal surface directly against the distal surface of the second molar, merely raising the gum but not coming through it. In one such case seen by the writer the patient had all the symptoms of an exposed pulp, but the closest examination could not discover one; eventually the third molar was found and the flap of gum was raised. It had presented as stated: a large cavity had formed in the occlusal surface through which the pulp had been exposed, thus confirming the diagnosis. Malposition of the upper third molar is, as a rule, back of and external to the tuberosity.

One exception to this coming under the writer's notice occurred in the mouth of a patient twenty-eight years old. She complained of neuralgic pains all over the side of the face and head, with marked soreness of the second molar, which had been devitalized two years previously. Thinking the tooth was beginning to abscess, I removed the filling and redressed the roots. No relief being obtained, the tooth was extracted, but the trouble was not relieved at once, and some other cause was suspected. As the pain was periodic, malarial influence was considered, and she was treated by her physician, but with little relief. In a few weeks the third molar came through in the place previously occupied by the second molar, and there has been no pain since.

Sex considerations are of importance in diagnostic work, inasmuch as symptoms vary according to the conditions under which they occur. Subjective symptoms in a neurotic female are valueless, or nearly so, for they magnify sensations to pains and pains to tortures, none the less real to them, but misleading to the clinician who is trying to arrive at accurate conclusions.

The period of catamenia is often accompanied by hemorrhagic conditions of the gums and tenderness of the teeth. Pregnancy often causes similar symptoms, and in the mouths of patients wearing artificial dentures much difficulty is experienced in retaining them, owing to lack of adaptation due to swelling of the mucous membranes. These cases are rather hard to manage unless the patient owns up. In one such case the writer had the patient denied being pregnant, but a few months confirmed the diagnosis.

Neuralgias and phantom odontalgias are especially frequent during pregnancy, and by recognizing this we may be saved the trouble of removing many good fillings in teeth which in themselves are without fault.

The state of the patient's health previous to or at the time of consultation may be of great importance as a direct or predisposing cause or as a modifying influence. An attack of la grippe is almost sure to give rise to dental discomforts, sometimes followed by the death of one or more pulps. In the case of one patient noted by the writer, after an unusually severe attack of this malady, three pulps died in a few weeks, one in a perfectly sound incisor. Influenza or cold in the head causes increased sensitivity of dentine and sometimes general tenderness and soreness of the whole denture.

Specific diseases do not, as a rule, affect the dental organs except through medication. The administration of the mercurials sometimes causes salivation or mercurial ptyalism, with its attendant ills, varying from slight looseness of the inferior incisors, with increased flow of saliva and slight metallic taste, to partial loss of the inferior maxilla, with adhesions of the tongue and cheek, and in very extreme cases death. The amount of mercury taken need not influence diagnosis, as some patients are idiosyncratically opposed to the mercurial treatment.

Lithiasis or gouty diathesis is now recognized as the predisposing, if not the direct, cause of most cases of pyorrhœa alveolaris.

Chronic catarrhal disease of the nose and sinuses may be the cause of, or be associated with, antral disease, and should be closely considered in making a differential diagnosis where dental complications are suspected.

Gangrenous stomatitis is often accompanied by ulcerous patches in the mouth, which lose much of their significance if their cause and association is recognized.

Prolonged illness, such as is caused by low forms of fever, bowel perforations, etc., which compel a fluid or semi-solid diet, cause general tenderness of the dental organs through lack of use, the peridental membrane becoming so tender that the slightest effort at mastication causes pain.

Recognized neurosis and neurasthenia is of value negatively, keeping us from putting too much credence in the testimony of the patient, cautioning us to be guarded in our statements, and admonishing us never to see them without the presence of the third person, as they are liable to form a distorted or entirely wrong conception of what is said to them. In one such case coming under the writer's notice the dentist casually remarked that he feared the patient (a lady) would lose her teeth from pyorrhœa, they were so dense and seemed so perfect; in fact, just such teeth as were liable to suffer from that disease. The patient went direct to a painless emporium and had the upper denture extracted forthwith, claiming she was so advised by her dentist. The husband threatened a suit for damages, but was dissuaded by the family physician. While the dentist was not wholly to blame, he was in a sense partially so, as he should have been able to recognize the mental limitations of a neurotic patient. Care should be used also in speaking of other practitioners before these patients, for they can never hear things straight; nor should we listen to the tales they bring us from other dentists. As smoke is to the eyes, or as vinegar to the taste, so is a neurotic patient in the experience of a dentist.

Occupation need be considered only in a general way. We would naturally refrain from using large metal fillings in the teeth of an "upholsterer" or "shoe-laster" unless the fillings had been well insulated. When a patient presents with his upper and lower incisors worn to the form of a circle, we know we are dealing with a "glass-worker," and before we repair the damage already done we explain that he must hold his blow-pipe against his lips and not between his teeth; or, better still, we swage up metal caps to be worn over the teeth during his work-hours. The lady with checked and broken incisors is catalogued as a "seamstress," and we are on our guard for exostosed roots or dead pulps. The phosphor-worker and the looking-glass plater are specially noted for their liability to phosphor necrosis and mercurial salivation. Patients working in chemical laboratories, gunpowder-works, or other places where they are subjected to the fumes of nitric, sulphuric, or muriatic acids, especially if mouth-breathers, seldom are able to preserve their dental organs without crowning.

In many ways habit and occupation could be treated under the same heading, especially in cases where trouble is the result of mechanical irritation such as is caused by thread-biting, the holding of mechanical appliances, etc., with the teeth, all of which are liable to cause pathological conditions, such as death of the pulp, exostosis, or absorption of permanent roots. Aside from these there are habits which might be termed vices, such as thumb-sucking, which causes anterior protrusion and lateral contraction; gritting the teeth at night, causing the undue wearing of the dental organs due to reflex nerve irritation, usually intestinal; mouth-breathing, due either to polypi, adenoid growths, or congenital defects. Cracking ice between the teeth is a prolific cause of dead pulps, especially in the mouths of children. It is noticed also in the mouths of patients convalescing after typhoid, where they have been given pieces of ice to eat when partially delirious.

Tobacco chewing, though often spoken of as beneficial to the teeth, is, in the mind of the writer, open to question, both because of increased attrition and because of a tendency to erosion on the buccal surfaces of those teeth in that part of the mouth where the tobacco quid is held. It is, at least, not conducive to personal cleanliness, as we dentists know.

Accidents are the direct cause of many dental troubles and the indirect cause of not a few, and, since "accidents are bound to occur," we must give them consideration. Accidents may properly be divided into two classes,—the unavoidable, those which happen with us, and the ones due to carelessness, or those which happen with others. It might be best to speak only of the latter.

Broken jaws improperly set or dislocations not reduced are the cause of various deformities. One of the most interesting cases known to the writer was that of a young man of eighteen, who called for treatment of a lower molar. There was a marked protrusion of the lower jaw, which was also forced to one side. Being impressed with the appearance of the case, I questioned the patient as to previous injury, when he disavowed any knowledge of any, and so did his mother, until, on second thought, she said she did remember that when he was a baby he was out where the men were milking and was kicked over and stepped on by a cow. The only inconvenience noticed at the time was a slight swelling and a reluctance to take the bottle, but nothing was thought of it.

Roots broken in attempted extraction are a common and easily detected form of trouble. Roots perforated in opening up the root-canals are a serious form of trouble, and where the perforation is near the apex it is not easy to distinguish it from a large foraminal opening, the chief diagnostic point being the character and flow of the hemorrhage.

Unbanded crowned roots which have split under stress sometimes present the appearance of true abscess. The best diagnostic point is the blueness and swelling of the gum extending to the gingival border, with tenderness confined to the affected tooth, and lack of pulsing or throbbing. Broken toothpicks, particles of filling-materials, retained ligatures, bristles from tooth-brushes, etc., need to be carefully sought for in all cases of local irritation; malocclusion caused by excess of filling-material may cause pericementitis. A broken broach or root-filling material forced through the foraminal opening may cause continued irritation until removed. In most cases diagnosis must be differential, except where the root can be entered with a Gates-Glidden drill; in such cases a magnet applied to the cuttings will attract small particles of the steel where the drill has come in contact with the broken instrument and cut it. Another diagnostic point is the slowness of the tooth to abscess or its refusal to do so unless septic conditions are present.

Carelessly placed arsenical applications are responsible for serious complications, which may be easily recognized by the appearance of the gum and the history of the case.

Temperament and idiosyncrasy influence our conclusions less in making diagnosis than in the treatment of disease. It is well to remember, however, that in the bilious bases we may reasonably expect secondary dentine to protect pulps in cavities that in the lymphatic or sanguine bases would be exposed. We may expect pulp irritation in the nervous temperament in simple caries. The sanguine temperament will develop severe acute inflammations with slight cause, while in the teeth of the lymphatic we often find dead pulps in simple cavities without the history of a single pain.

Idiosyncrasies noted from the stand-point of the dental diagnostician are few, but pronounced. Occasionally we find patients who seem immune from arsenical impress. Some cannot indulge in a single dish of strawberries without suffering agonies from sensitive dentine; cocaine in the smallest doses will produce dangerous symptoms in some cases, and cannot be administered at all in others; morphia is so little used in dentistry that the dentist need scarcely consider the peculiar idiosyncrasy sometimes found in connection with it; mercurial idiosyncrasies have already been mentioned except in the cases of ptyalism caused by vulcanite dentures and amalgam fillings. These cases I leave to the discussion of my high-potency brethren, for I have yet to see my first case.

